# Genistein enhances the effect of cisplatin on the inhibition of non-small cell lung cancer A549 cell growth *in vitro* and *in vivo*

**DOI:** 10.3892/ol.2014.2597

**Published:** 2014-10-09

**Authors:** DEZHI LIU, LING YAN, LAN WANG, WEICHENG TAI, WEILI WANG, CHANGBIN YANG

**Affiliations:** Department of Radiation Oncology, The Tumor Hospital of Jilin Province, Changchun, Jilin 130012, P.R. China

**Keywords:** cisplatin, genistein, non-small cell lung cancer, lung cancer

## Abstract

Although cisplatin (DDP) has been reported to be a promising antitumor therapy for non-small cell lung cancer (NSCLC), the effectiveness of the treatment remains limited due to an inherent tumor resistance to DDP. Genistein (GEN) is an abundant, naturally occurring isoflavonoid found in soy products that has been demonstrated to increase the anti-neoplastic activity of certain chemotherapy drugs in multiple tumor types. In the present study, DDP in combination with GEN was selected as a potential treatment to suppress tumor growth and simultaneously reduce the doses of the two drugs required for the treatment of NSCLC. Cell growth inhibition, apoptosis, cell cycle distribution and receptor signaling assays were conducted. In the *in vivo* study, DDP and GEN, either alone or in combination, were used to treat a xenograft model of the A549 cells. It was found that the combination of low concentrations of DDP and GEN induced significantly greater growth inhibition (P<0.01) and increased apoptosis in the A549 cells compared with either agent alone. In addition, DDP in combination with GEN could significantly suppress tumor growth *in vivo* compared with either agent alone. Combination treatment significantly suppresses constitutive phosphorylation of AKT and phosphoinositide-3 kinase, which may contribute to the inhibition of tumor growth. Overall, the present data suggested that GEN can increase the anti-neoplastic activity of DDP and that a combination of GEN and DDP is a potential drug candidate for the treatment of NSCLC.

## Introduction

Lung cancer is one of the primary causes of cancer-associated mortality in numerous countries (1,2), with non-small cell lung cancer (NSCLC) being the cause of 80–85% of all diagnosed lung cancers (3). At the time of diagnosis, numerous patients with NSCLC already exhibit advanced stage IIIB or IV disease, which makes these patients candidates for systemic chemotherapy (4). Cisplatin (diamminedichloroplatinum; DDP) is a platinum-coordinated complex that has been extensively used for first-line chemotherapy to treat human NSCLC (5). However, drug resistance often develops in NSCLC following continuous infusion or multiple administration of DDP, leading to treatment failure characterized by tumor growth or tumor relapse (6,7). Therefore, it is extremely important to decrease the resistance to chemotherapeutic drugs in order to treat NSCLC.

Genistein (GEN) is a major isoflavone in soy and red clover that has potential beneficial effects and numerous biological actions, which have become a focus for research. An increasing number of studies have revealed that dietary isoflavones, including GEN, may protect against several cancer types, including lung cancer (8–10), without exerting toxic effects on normal cells. In addition, several studies have demonstrated that GEN inhibits the proliferation of cancer cells by cooperatively or synergistically enhancing the effect of anticancer drugs (8,11,12). Schabath *et al* (13) found that 10 μM GEN enhanced trichostatin A-induced apoptosis in human lung carcinoma A549 cells, but not in normal human lung fibroblasts. It has been reported that GEN may sensitize estrogen receptor-positive breast cancer cells to tamoxifen treatment (14). Mohammad *et al* revealed that in *in vitro* and *in vivo* pancreatic carcinoma cells, GEN enhanced DDP-induced growth inhibition and apoptosis (15). These studies indicate that GEN may cause other drug-resistant carcinoma cells to become sensitized to common chemotherapy drugs and may also decrease the toxicity of these common agents, as a lower dose of the chemotherapy drug is required.

Currently, the effect of combined GEN and DDP treatment on NSCLC cell proliferation and growth *in vitro* and *in vivo* has not been explored in any systemic study. The present study, therefore, aimed to evaluate the feasibility of using DDP in combination with GEN to inhibit NSCLC cell growth, proliferation and apoptosis, and to investigate the underlying molecular mechanisms involved in GEN-induced apoptosis, thereby providing additive or synergistic benefits against NSCLC.

## Materials and methods

### Reagents

All chemicals used were reagent grade or higher. DDP and GEN were purchased from Sigma-Aldrich (St. Louis, MO, USA). The nonidet P-40 lysis buffer, chemiluminescent peroxidase substrate, propidium iodide (PI), 4′,6-diamidino-2-phenylindole (DAPI), 3-(4,5-dimethylthiazol-2-yl)-2,5 diphenyltetrazolium bromide (MTT), pyrogallol and H_2_O_2_ were obtained from Merck (Whitehouse Station, NJ, USA). Dimethyl sulfoxide (DMSO) was purchased from Merck (Darmstadt, Germany). The stock solutions of PI, DAPI and MTT were prepared by dissolving 1 mg of each compound in 1 ml phosphate-buffered saline (PBS). The solutions were protected from light, stored at 4°C and used within one month.

### Cell culture and cell viability assay

The A549 cells were obtained from the American Type Culture Collection (Manassas, VA, USA) and were cultured in Dulbecco’s modified Eagle’s medium (DMEM; GIBCO-BRL, Grand Island, NY, USA) supplemented with 10% heat-inactivated fetal bovine serum (FBS; GIBCO-BRL) at 37°C in a 5% CO_2_ atmosphere and at 95% humidity.

The A549 cells were grown in monolayers and were harvested and dispensed in 96-well culture plates in 100 μl DMEM at a concentration of 5×10^3^ cells per well. After 24 h, the assigned concentrations of DDP, GEN or a combination of the two were added to the cells. Wells containing medium alone were used as the negative controls, while the wells containing cells but without any treatment were used as the positive controls. The cells were then incubated for an additional 4 h. At the end of the treatment, 200 μl DMSO was added to each well following the removal of the supernatant. The cell viability was determined by measuring the absorbance of the wells at a wavelength of 490 nm using the Thermo Multiskan MK3 microplate reader (Thermo Fisher Scientific, Inc., Waltham, MA, USA). This assay was performed in triplicate. The inhibition rate was calculated according to the following formula: Inhibition rate (%) = [1 - (average absorbance of experimental group / average absorbance of blank control group)] × 100.

### Detection of apoptosis

The A549 cells were cultured in six-well plates in DMEM supplemented with 10% FBS medium and were treated with DDP, GEN or DDP and GEN in combination for 48 h. The cover slips were washed three times with PBS and single cell suspensions were fixed in 1% PBS. The cells were stained with 100 μg/ml acridine orange (Sigma-Aldrich) and 100 μg/ml ethidium bromide (Sigma-Aldrich) for 1 min. The cells were then observed under a fluorescence microscope (BX43, Olympus Corporation, Tokyo, Japan). In total, ≥200 cells were counted and the percentage of apoptotic cells was determined. Triplicates were performed in all experiments and the experiments were performed on five occasions.

### Caspase activity

The activity of caspase-3, -8 and -10 was measured using caspase colorimetric protease assay kits (Millipore, Billerica, MA, USA) according to the manufacturer’s instructions. In brief, the A549 cells were treated with DDP and GEN alone or in combination for 24 h. Following treatment, the cells were washed twice with ice-cold PBS and harvested by centrifugation at 1,000 × g for 10 min. The cell pellets were then lysed in 150 μl buffer. Protein concentrations in the lysate were determined using the Lowry method (16). An 80-μl aliquot of the lysate was incubated with 10 μl of substrate for each caspase at 37°C for 2 h. The samples were analyzed at 405 nm in a microplate reader (Thermo Fisher Scientific Inc.). The relative caspase activity of the control group was set as 100.

### Tumor xenograft assay

Female BALB/c nude mice, 4–5 weeks old, were obtained from the Experimental Animal Center of the Jilin University (Changchun, Jilin, China). All the animal experiments were performed in accordance with institutional guidelines, following a protocol approved by the Ethics Committees of the Disease Model Research Center (The First Hospital of Jilin University, Changchun, Jilin, China). Female BALB mice, 6–7 weeks old, were maintained under specific pathogen-free conditions and provided with food and water *ad libitum*. All the animals were fed with a normal pellet diet for one week prior to the experiments.

The exponentially growing A549 cells were harvested and a tumorigenic dose of 2.5×10^6^ cells was injected intraperitoneally into the 4–5 week-old mice. When the tumors reached 100–200 mm^3^ in size, the mice were divided randomly into four groups, with 10 mice per group. The control group received 1% polysorbate resuspended in deionized water. In the DDP group, DDP was administered at a dose of 9 mg/kg as an intraperitoneal bolus injection, while in the GEN group, GEN was administered at a dose of 800 μg/kg each day orally for five days. In the combination group, DDP (5 mg/kg) was administered with GEN on day one, followed by four days of GEN (500 μg/kg) treatment. The tumor weight was measured subsequent to the mice being sacrificed by cervical dislocation, and the tumor volume was measured prior to the treatment injections being administered and on days seven, 14 and 21 of treatment.

### Western blotting

The cultured cells were washed twice with PBS (pH, 7.2) and the cells were then lysed with Triton X-100 9Sigma-Aldrich) in Hepes buffer (Sigma-Aldrich), which consisted of 150 mm NaCl, 50 mm Hepes, 1.5 mm MgCl_2_, 1% Triton X-100, 0.1% SDS, protease inhibitor cocktail, 100 mm NaF and 100 mm Na_3_VO_4_, for 30 min. The cell lysates were clarified using centrifugation at 10,000 × g for 15 min and the protein concentrations were determined using the Bradford reagent (Sigma-Aldrich). The protein samples were separated on an 8–15% SDS-polyacrylamide gel and transferred onto nitrocellulose membranes (Santa Cruz Biotechnology, Inc., Dallas, TX, USA). Subsequent to blocking in Tris-buffered saline containing 5% skimmed milk and 0.1% Tween 20 for 2 h at room temperature, the protein samples were then immunoblotted with primary mouse monoclonal anti-human PI3K (1:3,000), anti-human phosphorylated(p) PI3K(p-PI3K; 1:2,000), anti-human AKT (1:3,000), anti-human pAKT (1:3,000) and anti-human β-actin (1:5,000) antibodies (Abs), which were all purchased from Cell Signaling Technology, Inc., (Beverly, MA, USA) for 2 h at room temperature. Next, the samples were incubated with horseradish peroxidase-conjugated polyclonal goat anti-mouse immunoglobulin G Abs (1:5,000, Amersham Biosciences, Uppsala, Sweden) for 2 h at room temperature. All the immunoblots were visualized using an enhanced chemiluminescence kit (Pierce Protein Biology, Rockford, IL, USA).

### Statistical analysis

The data are expressed as the mean ± standard deviation. Statistical comparisons between more than two groups were performed using one-way analysis of variance followed by Tukey’s post-hoc test. Statistical analyses were undertaken using the SPSS^®^ statistical package, version 19.0 (SPSS Inc., Chicago, IL, USA) for Windows^®^ and GraphPad Prism version 5.01 (GraphPad Software, San Diego, CA, USA). P<0.05 was considered to indicate a statistically significant difference.

## Results

### Effects of DDP and GEN alone or in combination on A549 cell proliferation

**Figure 1:**
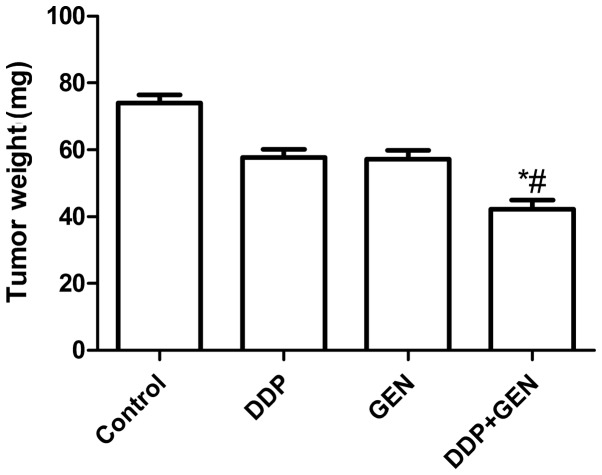
Effect of DDP and GEN alone or in combination on the inhibitory rate of A549 cells. The inhibitory rate of the A549 cells was determined by the MTT assay. Data are expressed as the mean ± standard deviation. ^*^P<0.05 vs. control; ^#^P<0.05 vs. DDP alone. DDP, cisplastin; GEN, genistein.

To evaluate the effect of DDP and GEN alone or in combination on the cell viability of NSCLC cells *in vitro*, a MTT assay was performed for 48 h whilst the A549 cells were being treated with DPP and GEN alone or in combination. It was found that the inhibitory rates of DDP and GEN alone or in combination were higher compared with the control group (P<0.01). There was no significance different between the DDP and GEN groups (P>0.05). However, the inhibitory rate in the group treated with GEN and DDP in combination was higher compared with either agent alone (P<0.05; Fig. 1).

### Effects of DDP and GEN alone or in combination on A549 cell apoptosis

**Figure 2:**
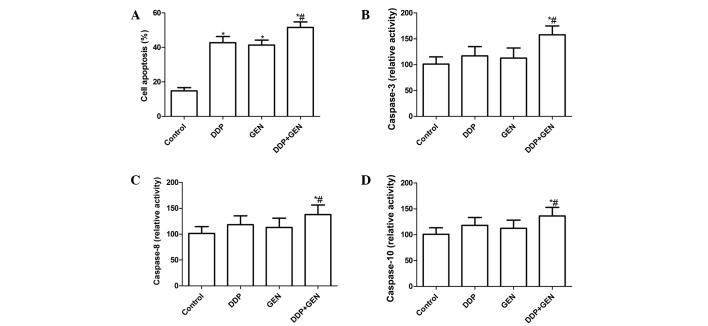
Individual or combined effects of DDP and GEN on cell apoptosis and caspase activity in A549 cells. (A) A549 cell apoptosis was determined 48 h after treatment with DDP and GEN alone or in combination. The individual or combined effects of DDP and GEN on (B) caspase-3, (C) caspase-8 and (D) caspase-10 activity in A549 cells is shown. ^*^P<0.05 vs. control; ^#^P<0.05 vs. DDP alone. DDP, cisplatin; GEN, genistein.

To investigate whether DDP and GEN alone or in combination could induce apoptosis, apoptosis was analyzed following treatment with DDP and GEN. It was found that the A549 cells treated with DDP or GEN had significant levels of cell apoptosis compared with the untreated cells (Fig. 2A). Treatment with a combination of DDP and GEN led to a marked increase in the level of apoptotic cells compared with either agent alone (P<0.01; Fig. 2A).

To explore the possible mechanism for the induction of cell apoptosis in cells treated with a combination of DDP and GEN, the activity of caspase-3, -8 and -10 was determined by ELISA. The results revealed that treatment with a combination of DDP and GEN could significantly increase the activity of caspase-3, -8 and -10 compared with treatment using DPP or GEN alone (P<0.01) (Fig. 2B, C and D).

### Treatment with DDP and GEN in combination causes significant inhibition of tumor growth

**Figure 3:**
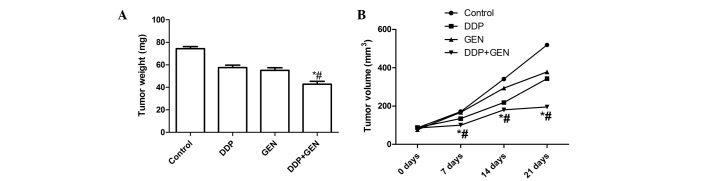
Antitumor activity of DDP and GEN in BALB/c mice bearing A549 tumors. (A) Tumor weight in treated and untreated mice after 21 days. (B) Tumor volume in treated and untreated mice on days 7, 14 and 21. Data are expressed as the mean ± standard deviation. ^*^P<0.05 vs. control; ^#^P<0.05 vs. DDP alone. DDP, cisplatin; GEN, genistein.

The *in vivo* therapeutic efficacy of DDP and GEN was assessed in female BALB/c nude A549 tumor-bearing mice. The mice were sacrificed and the tumor tissue was removed 21 days after treatment. The tumor weight was then measured and it was found that the tumor weight in the mice treated with a combination of DDP and GEN was lower compared with the tumor weight in the untreated group and the groups treated with either agent alone (P<0.01) (Fig. 3A). In addition, it was also found that the tumor volume following treatment with DDP and GEN in combination was significantly lower compared with the untreated group and the groups treated with either agent alone (P<0.01; Fig. 3B). These results indicate that treatment with DDP in combination with GEN markedly suppresses the tumorigenicity of A549 cells in mice.

### GEN in combination with DDP suppresses the PI3K/AKT pathway in A549 cells

**Figure 4:**
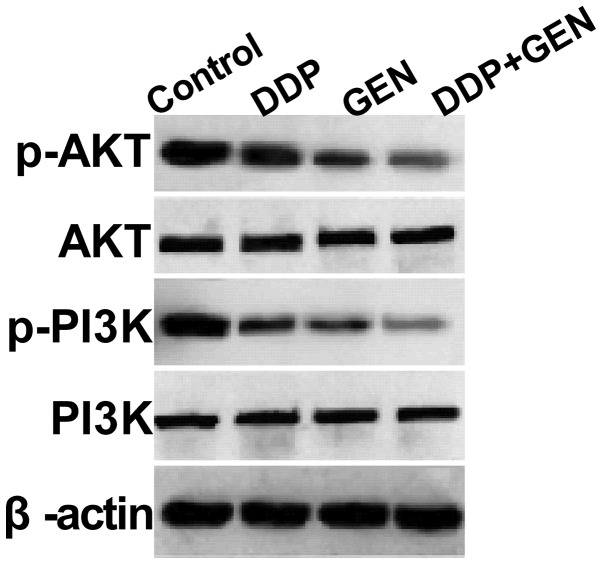
Individual or combined effects of DDP and GEN on the PI3K/AKT signaling pathway in A549 cells. The cells were treated with DDP and GEN alone or in combination for 24 h. Western blot analysis was performed using specific antibodies against the indicated proteins. DDP, cisplatin; GEN, genistein; PI3K, phosphoinositide 3-kinase; p-AKT, phosphorylated AKT; p-PI3K, phospho-PI3K.

To clarify the molecular mechanisms involved in the effect of GEN in combination with DDP on A549 cell proliferation, the present study focused on the effects of GEN and DDP alone and in combination on the activation of PI3K/AKT pathway, which participates in the main intracellular signaling required for cell proliferation and survival. GEN and DDP alone or in combination were found to inhibit the tyrosine phosphorylation of AKT and PI3K (Fig. 4). In addition, treatment with a combination of GEN and DDP resulted in a greater reduction in p-AKT and PI3K compared with treatment with either agent alone. These results indicate that treatment with DDP in combination with GEN inhibits tumor cell growth, to a certain extent, by suppressing the PI3K/AKT pathway.

## Discussion

Despite the substantial effort made to increase the survival rate of patients with NSCLC, the majority of patients exhibit advanced-stage, unresectable disease at the time of diagnosis and the lesions possess inherent resistance to chemotherapy and radiotherapy. Therefore, a satisfactory survival rate has not been reached. Administered as either a single drug or as a combination therapy with other agents, DDP is one of the chemotherapeutic agents used in the clinic to treat lung carcinoma (17). A previous study has suggested an association between DDP-based doublet regimens and a slightly improved survival rate compared with non-platinum-based doublet regimens (18). However, the outcome of DDP therapy for NSCLC appears to have reached a plateau. Therefore, the resistance to DDP must be decreased for the treatment of NSCLC.

In the present study, DDP in combination with GEN was selected to reduce the doses of DDP required for the treatment of NSCLC. The present results revealed that the combination of low concentrations of DDP and GEN resulted in significantly increased growth inhibition (P<0.01) and an increased level of apoptosis in the A549 cells compared with either agent alone. These findings suggested that GEN is able to increase the anti-neoplastic activity of DDP and decrease the inherent resistance of NSCLC to DDP.

GEN is a small, biologically active flavonoid that is found in high amounts in soy products and in other plants and vegetables. GEN is able to inhibit tumor cells *in vitro* and *in vivo* without evident toxicity to normal cells (19–21), making GEN a promising agent for use in conjunction with toxic chemotherapy agents, including DDP. It has been reported that GEN enhances DDP-induced cell growth inhibition and apoptosis in pancreatic and ovarian carcinoma cells (15,22). A previous study has also revealed that pretreatment with GEN inactivated NF-κB and may have contributed to an increase in DDP-induced growth inhibition and apoptosis in various cancers, without exhibiting systemic toxicity (23). The results of the present study revealed that GEN enhances DDP-induced cell growth inhibition and apoptosis in NSCLC cells *in vitro* and *in vivo,* which is in agreement with previous results (15,22,23). These findings and the present results demonstrate that GEN decreases the inherent resistance to DDP and increases the inhibition of tumor growth.

Caspases are members of a cysteine protease family that consists of integral components of the apoptotic pathway, including caspase-3. Caspase-3 is a cell death protease that is activated by a variety of apoptotic stimuli. Once activated, caspase-3 acts on numerous cellular targets that induce the morphological appearance of apoptotic cells when they are cleaved or activated (24,25). A large number of studies have concluded that numerous chemotherapy drugs exert apoptotic effects through the activation of caspases (24–26). In the present study, the results revealed that treatment with a combination of DDP and GEN significantly increased the activity of caspases-3, -8 and -10 compared with treatment with DDP or GEN alone (P<0.01), which indicates that caspases are vital protease mediators of apoptosis initiated by combined treatment with DDP and GEN.

The PI3K/AKT pathway is known to play a significant role in regulating the chemoresistance of cancer cells (27–29). In addition, Li and Sarkar also demonstrated that GEN inhibits NF-κB activation, which is partly mediated through the AKT signaling pathway (30). A study by Park and Seol also revealed that the combination of GEN and TRAIL enhanced TRAIL-induced apoptosis in A549 cells by regulating the PI3K/AKT pathway (31). In the present study, the results revealed that the use of DDP in combination with GEN as a treatment for A549 cells resulted in a marked reduction of phosphorylated PI3K and AKT relative to the untreated cells, without altering the total protein levels of PI3K or AKT. Previous studies and the present results have revealed that the combination of GEN and DDP enhanced DDP-induced apoptosis in A549 cells by regulating the PI3K/AKT pathway, at least in part.

In conclusion, the present study reveals that DPP in combination with GEN could enhance the anti-proliferative and pro-apoptotic effects on NSCLC cell via effects on the anti-apoptotic PI3K/AKT signaling pathways, at least in part. Thus, it may be worthwhile to consider further evaluating the combination treatment for NSCLC in clinical trials.
